# The effector function of mucosal associated invariant T cells alters with aging and is regulated by RORγt

**DOI:** 10.3389/fimmu.2024.1504806

**Published:** 2024-11-28

**Authors:** Zhi Yang, Banxin Luo, Minhuan Li, Ziyun He, Chuanfu Ren, Xin Chen, Xing Kang, Hong Chen, En Xu, Wenxian Guan, Xuefeng Xia

**Affiliations:** ^1^ Department of General Surgery, Nanjing Drum Tower Hospital, The Affiliated Hospital of Nanjing University Medical School, Nanjing, China; ^2^ Department of General Surgery, Nanjing Drum Tower Hospital, Drum Tower Clinical Medical College, Nanjing University of Chinese Medicine, Nanjing, China; ^3^ Department of Andrology, Drum Tower Clinical Medical College of Nanjing Medical University, Nanjing, China; ^4^ Department of Gastrointestinal Surgery, Zhongnan Hospital of Wuhan University, Wuhan, China; ^5^ Department of General Surgery, Drum Tower Clinical Medical College of Nanjing Medical University, Nanjing, China; ^6^ Department of General Surgery, Taikang Xianlin DrumTower Hospital, The Affiliated Hospital of Wuhan University Medical School, Nanjing, China

**Keywords:** mucosal-associated invariant T cells (MAIT), MAIT1, MAIT17, aging, scRNA-seq (single-cell RNA sequencing)

## Abstract

**Introduction:**

Mucosal-associated invariant T (MAIT) cells are a predominant subset of innate-like T cells in humans, characterized by diverse gene expression profiles and functional capabilities. However, the factors influencing the transcriptomes and effector functions of MAIT cells, particularly at mucosal barriers, remain largely unclear.

**Methods:**

In this study, we employed single-cell RNA sequencing (scRNA-seq) and functional assays to investigate the transcriptomic and functional characteristics of intestinal MAIT cells in mouse models during aging. We also extended scRNA-seq analysis to human intestinal MAIT cells to compare their gene expression patterns with those observed in aged mice.

**Results:**

Our findings demonstrated that the transcriptomes and functional capabilities of intestinal MAIT cells shifted from MAIT17 to MAIT1 profiles with aging in mouse models, with notable changes in the production of cytotoxic molecules. Further scRNA-seq analysis of human intestinal MAIT cells revealed a segregation into MAIT1 and MAIT17 subsets, displaying gene expression patterns that mirrored those seen in aged mouse models. The transcription factor RORγt was expressed in both MAIT1 and MAIT17 cells, acting to repress IFNγ production while promoting IL17 expression. Moreover, reduced expression of RORC and Il17A was correlated with poorer survival outcomes in colorectal cancer patients.

**Discussion:**

These results suggest that aging induces a functional shift between MAIT1 and MAIT17 cells, which may be influenced by transcriptional regulators like RORγt. The observed alterations in MAIT cell activity could potentially impact disease prognosis, particularly in colorectal cancer. This study provides new insights into the dynamics of MAIT cell responses at mucosal barriers, highlighting possible therapeutic targets for modulating MAIT cell functions in aging and disease.

## 1 Introduction

Mucosal-associated invariant T cells (MAIT cells) are the predominant subset of innate-like T cells in humans (1). They recognize intermediates of riboflavin synthesis, a conserved metabolic pathway found in bacteria and fungi (2–6). MAIT cells exhibit characteristics of both innate and adaptive immunity (1). Unlike conventional T cells, which differentiate into various effector subsets following activation, MAIT cells acquire specialized effector functions during early development in the thymus and are transcriptionally poised for these functions upon egress (7, 8).

Previous studies indicate that MAIT cells possess diverse functional capabilities. Mouse and human MAIT cells include at least two subsets: IFNγ-producing MAIT1 cells and IL-17A-producing MAIT17 cells (9–16). In addition to IFNγ and IL-17, MAIT cells can produce a variety of other effector molecules, including various cytotoxic factors (5, 17–23). However, the specific effector molecules produced by MAIT1 and MAIT17 subsets remain incompletely understood. The factors influencing the gene expression profiles and functional capabilities of MAIT cells, as well as the correlation between their effector functions with disease prognosis, are still largely unknown.

Aging is a complex process associated with an increased incidence of cancers and other disorders. Aging is paradoxically associated with both a decline in adaptive immunity (immunoscencence) and elevated basal levels of inflammation (Inflammaging). Aging leads to deterioration in the adaptive immune system, characterized by diminished naïve T cell and B cell pools, impaired antibody responses, and a contracted TCR repertoire (24). Concurrently, abnormal activities of various innate immune cells and the accumulation of senescent cells contribute to chronic tissue inflammation (25, 26). However, the effects of aging on non-traditional T cells, such as various innate-like T cells enriched at mucosal barriers, remain poorly understood. Investigating the impact of aging on innate-like T cells particularly MAIT cells may provide novel insights into lymphocyte aging and reveal strategies to combat aging-associated disorders including malignancies.

Colorectal cancer (COAD) is a significant global health burden. The association between MAIT cell gene expression profiles with COAD remained unexplored. In addition, the incidence of COAD increases with age COAD (27), and the elderly individuals often face challenges in optimizing treatment strategies. Recent research has identified several genes associated with cellular aging that correlate with COAD prognosis (28). However, the precise impact of immune cell aging on COAD remains largely uncharacterized.

In this study, we report that aging induces a shift in both the transcriptomes and functional capabilities of MAIT cells, changing from MAIT17 to MAIT1 profiles. We further demonstrate that human and mouse MAIT cells share similar gene expression patterns for the MAIT1 and MAIT17 profiles. Our findings reveal that the transcription factor RORγt promotes IL17A production while repressing IFNγ production in both mice and humans. In addition, decreased expression of *RORC* and *IL17A* correlates with worse prognosis in colon cancers. These results provide insights into innate lymphocyte aging and may inform strategies to address aging-associated disorders such as cancer.

## 2 Materials and methods

### 2.1 Mice

Young (6-8 weeks) and aged (20 months) C57BL/6 mice of both sexes were bred in the animal research facility. All animal experiments were conducted in accordance with protocols approved by the Institutional Animal Care and Use Committee of Nanjing Drum Tower Hospital.

### 2.2 Human tissues

De-identified intestinal tissues were collected at Nanjing Drum Tower Hospital. Relative normal tissues from patients with colorectal cancers were obtained and stored in cold SPS-1 storage buffer before immediate processing. De-identified samples from 7 individuals of middle age (45-65) and both sexes were obtained. The study was approved by the Ethics Committee of Nanjing Drum Tower Hospital.

### 2.3 Isolation of lymphocytes from tissues

For isolation of lymphocytes from mouse and human intestines, intestine tissues were cut into small pieces, and epithelial cells were dissociated with HBSS containing 5 mM EDTA, 1 mM DTT, and 2% FBS, shaken for 20 minutes at 37°C. This process was performed twice. After each step, samples were vortexed, rinsed with PBS, and the epithelial cell layer was discarded. The remaining tissue was digested in HBSS with 1 mg/mL Liberase TM (Roche), and 200 μg/mL DNase I (Roche) for 30 minutes at 37°C. Percoll centrifugation was performed to remove dead cells and debris. The resulting single cells were strained through a 70 μM mesh.

For isolation of lymphocytes from mouse lungs, euthanized mice were perfused by injecting 20 ml cold PBS into the right ventricle of the heart. The lung tissues were minced by a scissor, and digested in HBSS with 1 mg/mL Liberase TM, and 200 μg/mL DNase I for 30 minutes at 37°C. The cells were then filtered through a 70 μM mesh.

For isolation of skin lymphocytes, the ear pinnae were obtained and separated into ventral and dorsal sheets. The sheets were digested in RPMI 1640 media with 0.5 mg/mL DNase I, and 0.25 mg/mL Liberase TL (Roche) for 1 hour and 45 minutes at 37°C. After digestion, the skin sheets were homogenized, followed by Percoll centrifugation to remove dead cells and debris. The single cell suspension was then passed through 70-μm filters.

### 2.4 Flow cytometry and cell sorting

Cells were stained with MR1-5-OP-RU tetramers together with surface antibodies at room temperature for 30 minutes. MR1 tetramers were generated by tetramerizing biotinylated monomers loaded with 5-OP-RU (Immundex) using streptavidin conjugated to APC (Thermo), as previously described (3, 29). Antibodies for human samples include anti-CD45 (HI30, cat#MHCD4528, Thermo), anti-CD3 (OKT3, 317338, Biolegend), anti-CD161 (130-113-591, cat#130-113-591, Miltenyi Biotec), anti-RORγt (AFKJS-9, cat # 12-6988-82, Thermo). Antibodies for mouse samples included anti-CD45 (104, cat# 109820, Biolegend), anti-CD3 (145-2C11, cat#100326, Biolegend), anti-IL18R (P3TUNYA, 12-5183-82, Thermo), anti-CD90 (53-2.1, cat#140310, Biolegend), anti-RORγt,(B2D, cat# 12-6981-82, Thermo). Intracellular staining of RORγt was performed using FoxP3 Fix/Perm Kit (Thermo) according to the manufacturer’s instructions. Ki67 staining was performed using the PE Mouse Ki67 Staining Kit (BD Biosciences) according to the manufacturer’s instructions. Annexin V staining was performed with the PE Annexin V Apoptosis Detection Kit (BD Biosciences) following the manufacturer’s instructions. For fluorescence activated cell sorting (FACS), human MAIT cells were sorted by fluorescence activated cell sorting (FACS) as CD45+ CD3 + 5-OP-RU tetramer+, with CD161 included to assist with gating and to exclude debris and nonspecifically stained cells. Mouse MAIT cells were identified as CD45+ CD3 + 5-OP-RU tetramer+, with IL18R included for gating assistance and to exclude debris and nonspecifically stained cells.

### 2.5 Single cell RNA sequencing

For single cell RNA sequencing with mouse cells, MAIT cells were pooled and sorted from samples from 5 young or 5 aged mice. For single cell RNA sequencing with human cells, MAIT cells were pooled and sorted from samples from 7 different human donors. scRNA-seq libraries were prepared using the chromium 5’ single cell gene expression kit from 10x genomics according to the manufacturer’s instructions. Sequencing was carried out using a NextSeq 500 (Illumina). Analysis was carried out using Cellranger. Normalization of the data was achieved using the SCTransform function within Seurat. Cell clustering was performed using Uniform Manifold Approximation and Projection (UMAP). Feature plots, and violin plots were generated using normalized data. A Wilcoxon rank sum test was used as a statistical significance test.

### 2.6 Gene set enrichment analysis

The GSEA algorithm was utilized to access the enriched gene sets from differential genes. In brief, the differential gene expression of targeted two groups was calculated with the R package limma 3.56.2. Then the differential genes were preranked by log2FoldChange and subjected to the R package clusterProfiler 4.8.3. Results with p value<0.05, NES>1 and False discovery rate<0.25 were considered significant.

### 2.7 Reverse transcription‐quantitative PCR

For RT–qPCR analysis, mRNA was isolated from FACS sorted MAIT cells using either the RNeasy Plus Mini Kit (Qiagen), following the manufacturer’s protocols. Complementary DNA (cDNA) was synthesized using SuperScript II Reverse Transcriptase (Thermo). qPCR was carried out using ABI pre-optimized TaqMan probes. Probes for mouse genes included those to detect *Ifng* (Mm01168134_m1), *Tbx21* (Mm00450960_m1), *Ccl5* (Mm01302427_m1), *Ccl4* (Mm00443111_m1), *Il17a* (Mm00439618_m1), *Il22*(Mm01226722_g1), *Ccr6*(Mm99999114_s1), *Il23r*(Mm00519943_m1), *Gzmb* (Mm00442837_m1), *Nkg7*(Mm01205900_g1), *Prf1*(Mm00812512_m1), *Gzma* (Mm01304452_m1), and *Gapdh* (Mm99999915_g1). Gene expression levels were calculated relative to Gapdh for normalization.

### 2.8 Survival analysis

Survival analysis was conducted using RNA-seq Data data and clinical information from 446 colon cancer (COAD) patients, obtained from The Cancer Genome Atlas (TCGA). The primary objective was to investigate the correlation between gene expression and overall survival outcomes. To assess the prognostic significance of specific gene expressions, an univariate Cox proportional-hazards regression model was applied to each gene with the “coxph” function of the R package “survival”. The hazard ratios (HR) and corresponding 95% confidence intervals (CI) were calculated for both genes, with p-values indicating the significance of their association with survival. To dichotomize the patients into high-expression and low-expression groups for *IL17A* and *RORC*, the optimal cut-off points were determined by the “surv_cutpoint” function of the R package “survminer”. The cut-off values were selected based on the maximization of the log-rank statistic. Kaplan-Meier survival curves were plotted for the survival rates of high-expression and low-expression groups. The differences in overall survival between the two groups were statistically evaluated using the log-rank test.

### 2.9 *In vitro* culture of MAIT cells

For culturing of mouse MAIT cells, 96-well culture plates were bound with 3μg/ml of anti-CD3 (clone 2C11) and anti-CD28 (clone 37.51) antibodies overnight, before MAIT cell culture. 5000 mouse MAIT cells were sorted by FACS and cultured with DMEM medium supplemented with 1% ITS-G supplement (Thermo), 1% of penicillin and streptomycin, 100ng/ml IL-7 (R&D), in the presence of 5μM RORγt inhibitor GSK805. Cells were cultured for 3 days, and cell-free culture supernatant was collected. Concentrations, cytokines and chemokines were measured by bead-based multiplex assays using LegendPlex kits (Biolegend) according to the manufacterer’s instructions. Granzyme B concentrations were measured by ELISA (Thermo).

For culturing of human MAIT cells, 96-well culture plates were bound with 1μg/ml of anti-CD3 (clone OKT3) and 5μg/ml of anti-CD28 (clone CD28.2) antibodies overnight, before cell culture. 50,000 human MAIT cells were sorted by FACS and cultured with DMEM medium supplemented with 1% ITS-G supplement (Thermo), 1% of penicillin and streptomycin, 100ng/ml IL-7, and 5μM RORγt inhibitor GSK805 or vehicle control. Cells were cultured for 2-3 days and supernatant were collected. Concentrations of cytokines, chemokines and cytotoxic molecules were measured by bead-based multiplex assays using the LegendPlex kits (Biolegend).

### 2.10 Statistical analysis

Wilcoxon rank sum test was used to determine the significance of scRNA-seq data. Student T cells or one-way ANOVA was used for data obtained from other experiments. p<0.05 was considered significant.

## 3 Results

### 3.1 MAIT cells accumulate with aging in mice

**Figure 1:**
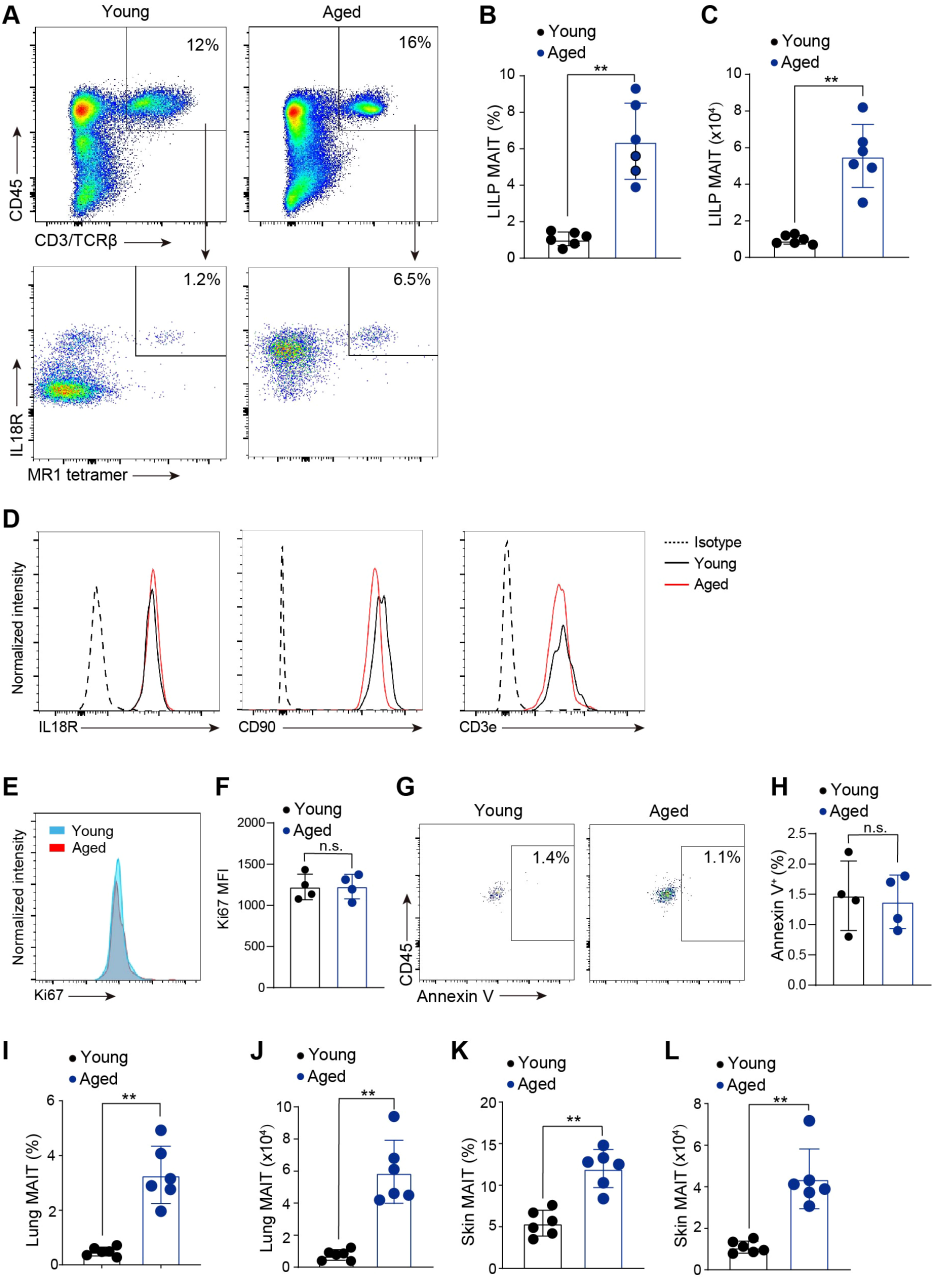
MAIT cells accumulated at mucosal barriers with age. **(A)** Representative flow cytometry flow profiles of MAIT cells in the large intestinal laminal propria (LILP) of young (6-8 weeks) and aged (20-month) mice. Plots were pre-gated on lung DAPI-CD45+CD3/TCRβ+ cells. **(B)** Percentages of MAIT cells in the LLIP of young and aged mice. **(C)** Numbers of MAIT cells in the LLIP of young and aged mice. **(D)** Representative flow cytometry profiles showing expression of IL7Ra, IL18R, and CD90 by intestinal MAIT cells in young and aged mice. **(E)** Representative flow cytometry profiles showing expression of Ki67 in MAIT cells from young and aged mice. **(F)** Mean fluorescence intensity (MFI) of Ki67. **(G)** Representative flow cytometry profiles showing Annexin V staining in MAIT cells from young and aged mice. **(H)** Percentages of MAIT cells with positive Annexin V staining. **(I)** Percentages of MAIT cells in the lungs of young and aged mice. **(J)** Numbers of MAIT cells in the lungs of young and aged mice. **(K)** Percentages of MAIT cells in the skin of young and aged mice. **(L)** Numbers of MAIT cells in the skin of young and aged mice. Data are from 4-6 mice per group, pooled from 2 independent experiments. ** p<0.01. ns, not significant.

Little is known about the effects of aging on innate-like T cells. To investigate the effects of aging on MAIT cells, we performed flow cytometry analysis to examine MAIT cells in the large intestinal laminal propria (LILP) of young (4-6 weeks old) and aged (20-month-old) mice. MAIT cells were identified as T cells that stained positively with MR1 tetramers. Because MAIT cells in mouse tissues express IL18R (30, 31), we used IL18R to help gate MR1 tetramer-staining positive cells and to exclude debris and cells with nonspecific tetramer binding (**Figure 1A**). Notably, MAIT cell percentages and abundance greatly increased with aging (**Figures 1A–C**). Our quantification of MAIT cells was based on the total MAIT cells present in the entire large intestine tissue from each mouse (**Figure 1C**). Because isolating lymphocytes from the intestines is a highly time-sensitive procedure, we were unable to measure tissue weight before digesting the samples. The identity of MAIT cells was validated by their high expression of characteristic surface markers including CD90, IL18R and IL7R (**Figure 1D**). Ki67 expression was comparable between MAIT cells from young and aged mice (**Figures 1E, F**). Annexin V staining revealed minimal apoptosis in MAIT cells isolated from both young or aged mice (**Figures 1G, H**). These results suggest that aging does not significantly affect the proliferation or survival of MAIT cells. Furthermore, a similar accumulation of MAIT cells was observed in the lung and the skin of aged mice, indicating that age-related increase in MAIT cells with aging is not restricted to intestines (**Figures 1I–L**). Together, these data indicate that MAIT cells accumulate at mucosal barriers with aging in mice.

### 3.2 The transcriptomes and functional capabilities of MAIT cells shift from a MAIT1 profile to MAIT17 profile with aging

**Figure 2:**
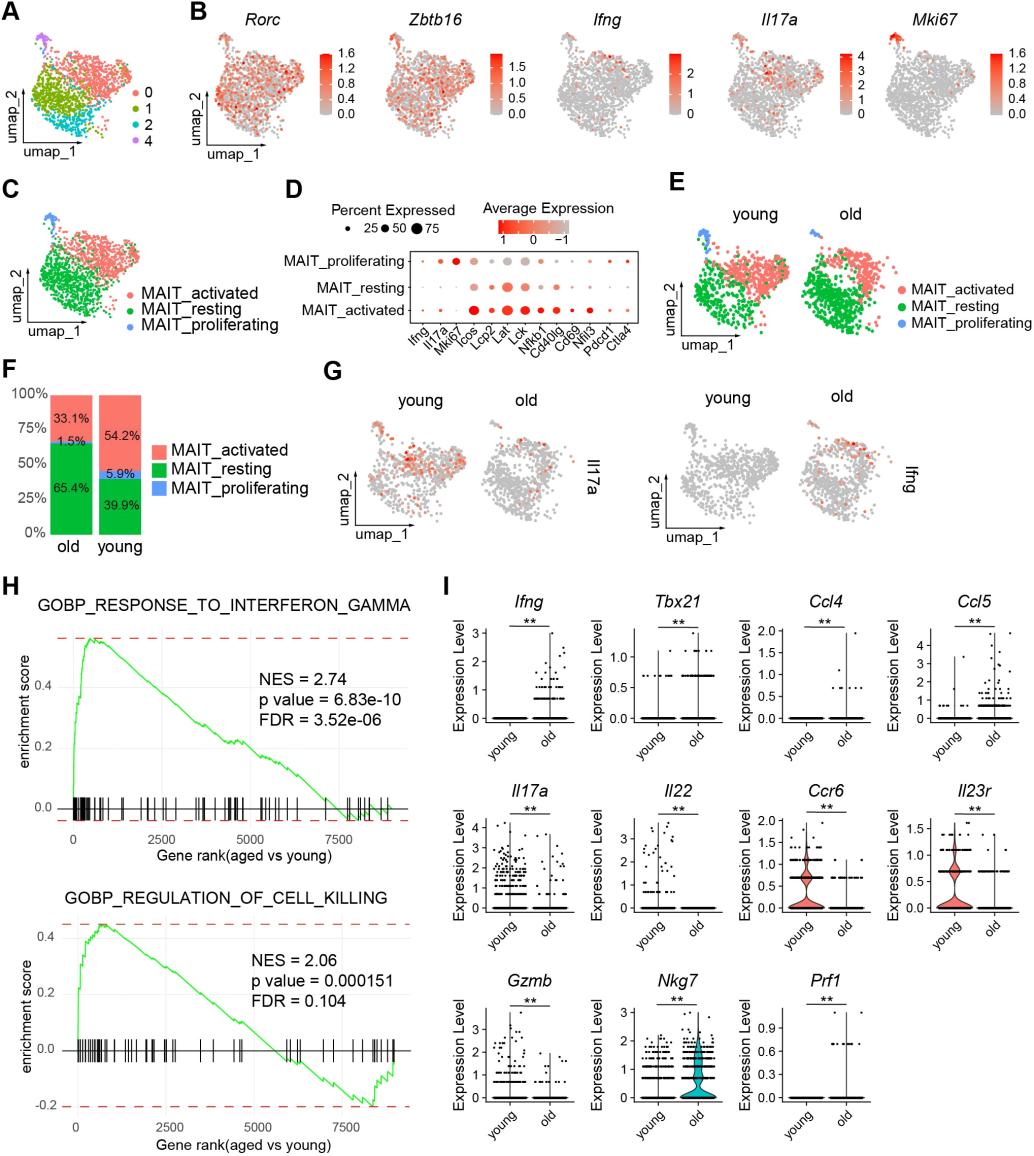
The transcriptomes of MAIT cells alter with age. Single cell RNA-seq (scRNA-seq) was performed with MAIT cells sorted from the large intestinal laminal propria (LILP) of young and aged mice. **(A)** UMAP profiles of the transcriptomes of intestinal MAIT cells. **(B)** Feature plots depicting expression of the indicated genes. **(C)** Annotation of MAIT cell subsets. **(D)**, Dot plots showing expression of the indicated genes by each MAIT cell subset. **(E)** UMAP profiles of MAIT cells from young and aged mice separately. **(F)** Percentages of each subset in MAIT cells from young and aged mice. **(G)** Feature plots showing expression of IFNG and IL17A by MAIT cells from young and aged mice. **(H)** Gene sets that are overrepresented in differentially expressed genes in MAIT_activated cells between young and aged mice. **(I)** Violin plots showing expression of the indicated genes by MAIT_activated cells from young and aged mice. Data are from 6 mice pooled per group. **p<0.01.

To examine the effects of aging on the gene expression profiles of MAIT cells, we performed single-cell RNA-sequencing (scRNA-seq) of MAIT cells sorted from LILP of young and aged mice (**Figure 2A**). The identity of MAIT cells was validated by their high expression of characteristic marker genes such as *Rorc* and *Zbtb16* (**Figure 2B**). Mouse intestinal MAIT cells were initially separated into four subsets by UMAP (**Figure 2A**). Subset 3 expressed high levels of proliferating gene markers such as *Mki67*, and were therefore annotated “MAIT_proliferating” (**Figures 2C, D**). Subset 1 and 2 exhibited similar transcriptome profiles, showing low expression of various lymphocyte activation gene (e.g. *Icos*, *Lcp2*, *Lck*, *Lat*, *Nfkb1, Cd40lg, Cd69, Nfil3*) and effector molecule genes including *Ifng* and *Il17a* (**Figures 2A, D**). These cells were thus annotated as “MAIT_resting” (**Figure 2C**). Subset 0 expressed high levels of genes associated with lymphocyte activation, and were thus annotated as MAIT_activated (**Figure 2C**). While gene positively regulate T cell activation (*Icos*, *Lcp2*, *Lck*, *Lat*, *Nfkb1, Cd40lg, Cd69, Nfil3*) were expressed at the highest levels in MAIT cells, checkpoint genes negatively regulate T cell activation (*Pdcd1, Ctla4*) were expressed at the highest levels in MATI_proliferating cells. Notably, MAIT cells from aged mice had a significantly higher average frequency of MAIT_resting cells compared to those from young mice, indicating that aging is associated with an accumulation of MAIT cells exhibiting an inactive phenotype. (**Figures 2E, F**). The frequencies of MAIT_activated and MAIT_proliferating cells were lower in MAIT cells from aged mice (**Figures 2E, F**). However, due to the overall increase in the total number of MAIT cells, the abundance of MAIT_proliferating was similar between young and aged mice, while the abundance of MAIT_activated increased with age. The increased percentage of resting MAIT cells in aged mice was also associated with decreased expression of several T cell activation genes (*Icos, Nfkb1, Cd40lg, Nfil3*) and checkpoint genes (*Pdcd1, Ctla4*) in the overall MAIT cell population (**Supplementary Figure S1**).

Interestingly, our scRNA-seq data revealed that MAIT cells from young mice primarily expressed *Il17a*, with minimal *Ifng* expression, while MAIT cells from aged mice showed a strong preference for *Ifng* (**Figure 2G**). Since the majority of cytokine-producing cells are found in the MAIT_activated subset, we focused our analysis on this group. GSEA indicated that gene sets associated with the interferon-gamma pathway and killer cell activity were significantly upregulated in MAIT_activated cells from aged mice compared to those from young mice (**Figure 2H**). MAIT_activated cells in aged mice exhibited markedly higher expression levels of *Ifng*, T helper type 1 (Th1)-related chemokines *Ccl5* and *Ccl4*, and the Th1 transcription factor *Tbx21* (encoding T-bet). In contrast, MAIT_activated cells from young mice expressed elevated levels of T helper type 17 (Th17)-related cytokines and receptors, including *Il17a*, *Il22*, *Ccr6*, and *Il23r* (**Figure 2I**). The expression of cytotoxic molecules varied with aging. Granzyme B (*Gzmb*) was more highly expressed in MAIT_activated cells from young mice, whereas the expression of other cytotoxic genes, *Nkg7* and *Prf1* (encoding perforin), was elevated in aged mice (**Figure 2I**). Thus, aging induced a shift from MAIT1 to MAIT17 profiles in MAIT cells, with variable effects on cytotoxic molecules.

**Figure 3:**
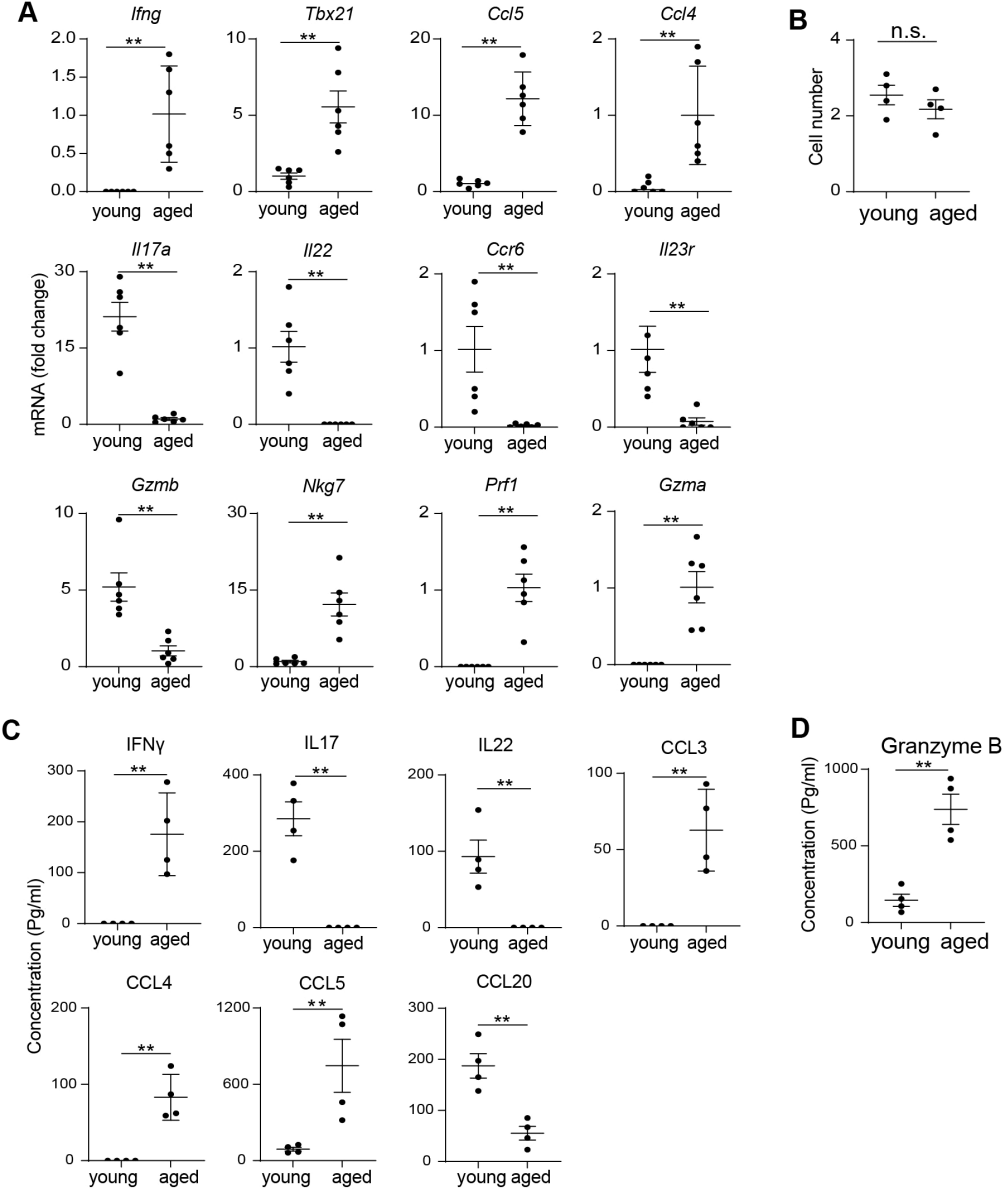
Aging is associated with a shift from MAIT17 to MAIT1 cells. **(A)** MAIT cells were sorted from the large intestinal laminal propria of young and aged mice by fluorescence activated cell sorting. mRNA levels of the indicated genes were measured by QPCR analysis. **(B)** 5000 sorted MAIT cell were cultured with IL-7 and plate-bound anti-CD3 and anti-CD28 antibodies for 3 days. Cell numbers after 3 days of culture were measured. **(C)** The concentrations of cytokines and chemokines in the culture supernatant were measured by bead-based multiplex assays. **(D)** The concentrations of granzyme B in the culture supernatant were measured by ELISA. Data are from 6 mice per group, pooled from 2 independent experiments **(A)**; or are from 4 mice per group, representative of 2 independent experiments **(B-D)**. QPCR data are normalized to Gapdh. **p<0.01.

To validate our scRNA-seq findings, we performed extensive QPCR analysis on MAIT cells sorted from individual mice. Post sorting flow cytometry analysis verified high post sorting purity (**Supplementary Figure S2**). QPCR results confirmed that MAIT cells from aged mice expressed higher levels of Th1-like cytokines and chemokines, including *Ifng*, *Ccl5*, and *Ccl4* (**Figure 3A**). In contrast, MAIT cells from young mice showed elevated expression of Th17-like cytokines and associated receptors, such as *Il17a*, *Il22*, *Ccr6*, and *Il23r* (**Figure 3A**). Additionally, the Th1-related transcription factor *Tbx21* was significantly upregulated in MAIT cells from aged mice compared to those from young mice (**Figure 3A**). While *Gzmb* was upregulated in MAIT cells from young mice, other cytotoxic genes, such as *Nkg7* and *Prf1*, were more highly expressed in MAIT cells from aged mice (**Figure 3A**). Although *Gzma* mRNA was barely detectable in the scRNA-seq data, it was identified in MAIT cells from aged mice but not from young mice through QPCR analysis (**Figure 3A**). Together, our results indicate that intestinal MAIT cells shift from a MAIT17 profile to a MAIT1 profile with aging, accompanied by decreased expression of *Gzmb* and increased expression of other cytotoxic molecules.

To assess whether the effector functions of MAIT cells change with aging, we sorted MAIT cells by FACS and cultured them *in vitro* with plate-bound anti-CD3 and anti-CD28 antibodies, adding IL-7 to enhance cell survival. Both MAIT cells from young and aged mice proliferated at similar rates *in vitro* (**Figure 3B**). We then measured the concentrations of cytokines and chemokines in the culture supernatants using Legendplex, a bead-based multiplex kit. Th1-like cytokines and chemokines, including IFNγ, CCL3, and CCL4, were readily detectable in the culture supernatant of MAIT cells from aged mice, but not from young mice (**Figure 3C**). CCL5 concentrations were also significantly higher in aged mice compared to their young counterparts (**Figure 3C**). In contrast, IL17 was prominently detected in the supernatants of MAIT cells from young mice, but not in those from aged mice (**Figure 3C**). CCL20, a chemokine associated with Th17 cells, was similarly present in young mice’s culture supernatants but absent in those from aged mice (**Figure 3C**). Additionally, MAIT cells from young mice produced higher levels of Granzyme B compared to those from aged mice, as measured by ELISA (**Figure 3D**). Other cytotoxic molecules were not assessed due to the unavailability of reliable ELISA or multiplex assay kits for those targets. Collectively, these data suggest that aging induces a functional shift in MAIT cells from a MAIT17 profile to a MAIT1 profile.

### 3.3 RORγt regulates the production of effector molecules by MAIT cells

**Figure 4:**
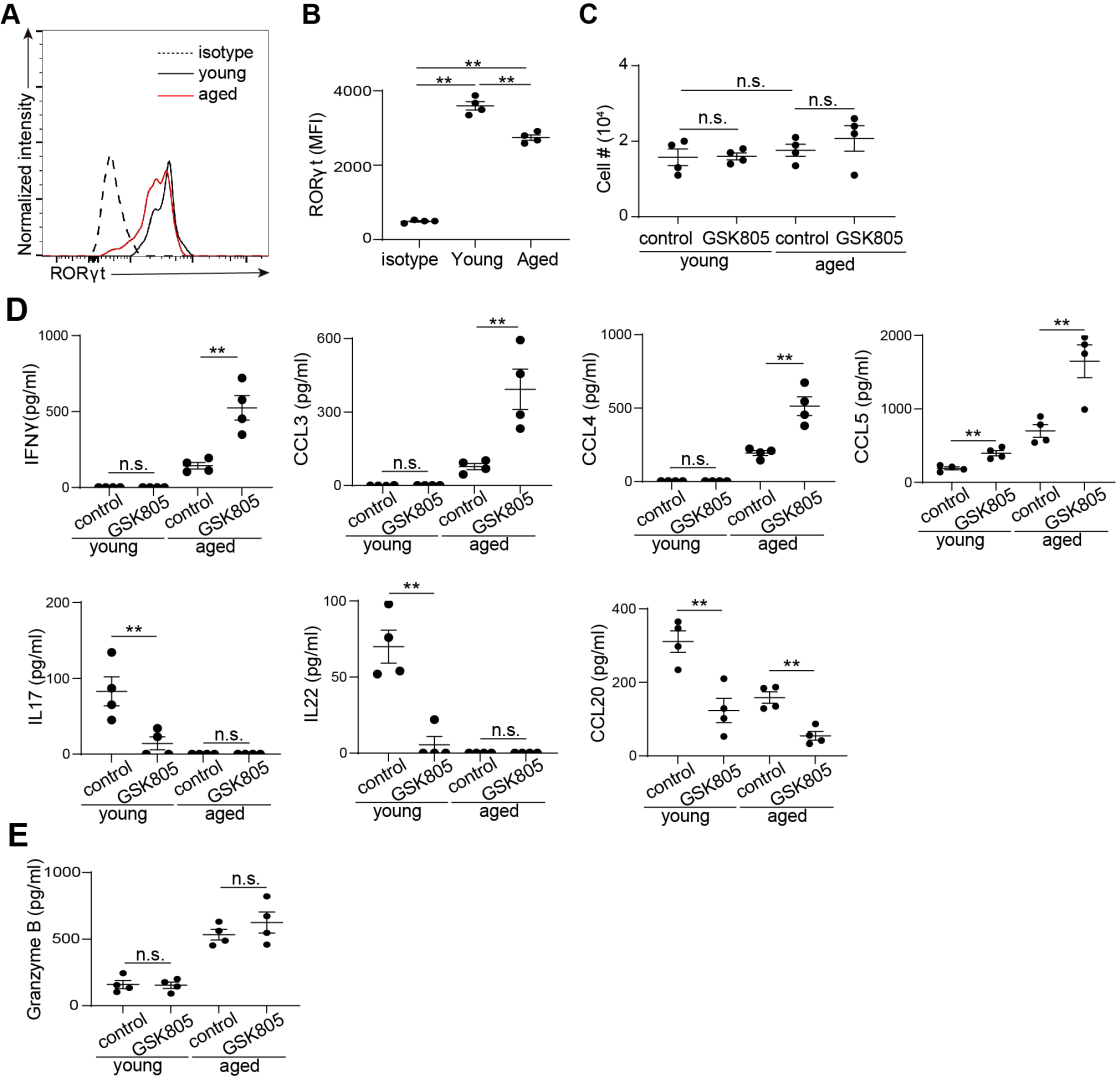
RORγt inhibition leads to reduced IL17 but increased IFNγ production in mouse MAIT cells. **(A)** Representative flow cytometry profiles showing expression of RORγt by by MAIT cells from the large intestinal laminal propria of young and aged mice. **(B)** Mean fluorescence intensity (MFI) of RORγt. **(C)** 5000 sorted MAIT cell were cultured with IL-7 and plate-bound anti-CD3 and anti-CD28 antibodies, in the presence of RORγt inhibitor GSK805 or vehicle control, for 3 days. Cell numbers after 3 days of culture were measured. **(D)** The concentrations of cytokines and chemokines in the culture supernatant were measured by bead-based multiplex assays. **(E)** The concentrations of granzyme B in the culture supernatant were measured by ELISA. Data are from 4 mice per groups, representative of two independent experiments. **p<0.01; n.s. not significant.

RORγt is a transcription factor highly expressed in MAIT cells (**Figure 2B**). Using flow cytometry analysis, we verified positive expression of RORγt in MAIT cells from both young and age mice (**Figure 4A**). However, the expression level of RORγt showed a moderate decrease with aging (**Figure 4B**). To investigate the role of RORγt in regulating MAIT cell function, we sorted MAIT cells by FACS and cultured them with the RORγt inhibitor GSK805 and vehicle control. Post sorting analysis verified high post sorting purity (**Supplementary Figure S3**). Inhibition of RORγt did not significantly affect MAIT cell growth *in vitro* (**Figure 4C**). However, we observed notable changes in cytokine and chemokine production (**Figure 4D**). Specifically, GSK805 significantly increased the production of Th1 cytokines and chemokines, including IFNγ, CCL3, CCL4, and CCL5, in MAIT cells from aged mice (**Figure 4D**). In contrast, the production of Th17-related cytokines and chemokines, such as IL17, IL22, and CCL20, in MAIT cells from young mice was diminished by GSK805 (**Figure 4D**). The production of Granzyme B remained unchanged with GSK805 treatment (**Figure 4E**). Together, these data indicate that RORγt promotes MAIT17 effector molecules while repressing MAIT1 effector molecules.

### 3.4 The transcriptomes of human intestinal MAIT cells are separated into MAIT1 and MAIT17 subsets

**Figure 5:**
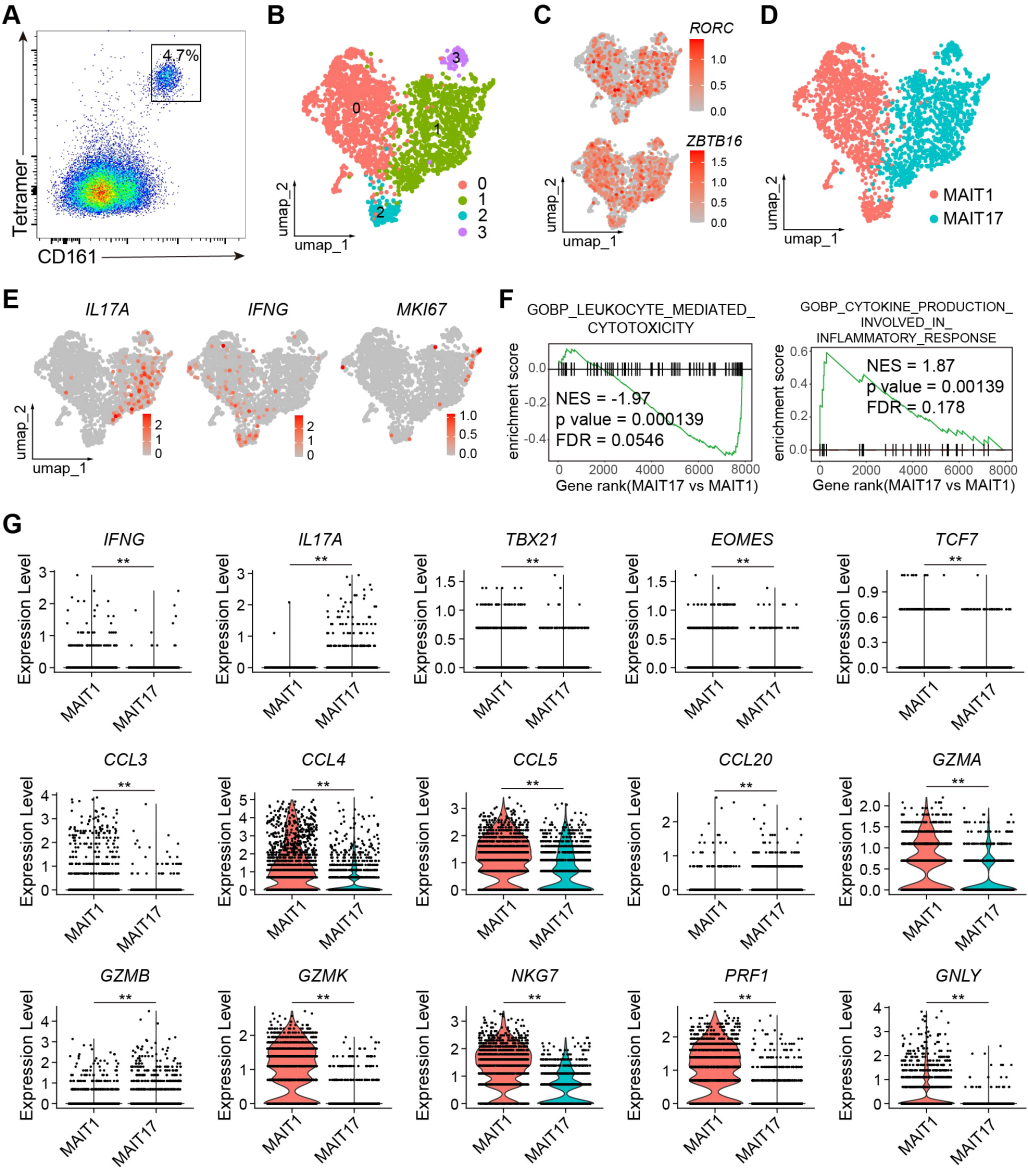
The transcriptomes of Human intestinal MAIT cells were separated into MAIT1 and MAIT17. **(A)** representative flow cytometry profile of human MAIT cells in human colon laminal propria. Plots were pre-gated on CD45+ CD3+ cells. **(B)** scRNA_seq were performed from human MAIT cells purified by FACS. Shown are UMAP plots. **(C)** Feature plots showing expression of RORC an ZBTB16 in human MIAT cells. **(D)** Annotation of human MAIT cells into MAIT1 and MAIT17 subsets. **(E)** Feature plots showing expression of the indicated genes. **(F)** Gene sets enriched in genes differentiability expressed between human MAIT17 and MAIT1 cells. **(G)** Violin plots showing expression of the indicated genes in human MAIT17 and MAIT1 cells. Data are from FACS-sorted and pooled MAIT cells from the large intestinal laminal propria of 7 human individuals. **, P < 0.01.

To examine the transcriptomes of human intestinal MAIT cells, we performed scRNA-seq on human colon laminal propria MAIT cells, identified using MR1 tetramers and characterized by high levels of CD161 (**Figure 5A**). Due to the effects of aging on MAIT cells, we obtained de-identified samples from seven individuals of middle age (45-65 years), to ensure inclusion of both MAIT1 and MAIT17 profiles. The identities of the MAIT cell transcriptomes were validated by high expression levels of the characteristic transcription factors *RORC* and *ZBTB16* (**Figures 5B, C**). UMAP analysis clearly separated MAIT1 and MAIT17 cells (**Figures 5D, E**). Subsets 0 and 2, which exhibited similar transcriptomes with elevated IFNG expression, were annotated as “MAIT1” (**Figures 5D, E**). Subsets 1 and 3, which were close at the transcriptomic level and showed high IL17A expression, were designated as “MAIT17” (**Figures 5D, E**). Proliferating MAIT cells did not form distinct clusters (**Figure 5E**). We then compared gene expression profiles between MAIT1 and MAIT17 cells. GSEA revealed that gene sets associated with lymphocyte cytotoxicity were upregulated in MAIT1 cells, while genes related to inflammatory cytokine responses were elevated in MAIT17 cells (**Figure 5F**). In addition to IFNG, MAIT1 cells exhibited higher levels of Th1-related chemokines, including CCL3, CCL4, and CCL5 (**Figure 5G**). Conversely, MAIT17 cells expressed higher levels of Th17-related cytokines and chemokines, such as *IL17A* and CCL20. MAIT17 cells also showed increased expression of *GZMB*, whereas MAIT1 cells expressed higher levels of other cytotoxic genes, including *GZMA*, *NKG7*, *PFR1*, and *GNLY* (**Figure 5G**). Furthermore, MAIT1 cells expressed higher levels of the Th1 characteristic transcription factor TBX21 and the cytotoxic lymphocyte transcription factor *EOMES* (**Figure 5G**). *TCF7* expression was also higher in MAIT1 cells compared to MAIT17 cells (**Figure 5G**). These gene expression patterns closely resemble the aging-related changes observed in mouse MAIT cells. Together, these findings indicate that the transcriptomes of human intestinal MAIT cells separated into MAIT1 and MAIT17 subsets, exhibiting expression patterns of effector molecules similar to those seen in aging-related changes in mouse MAIT cells.

**Figure 6:**
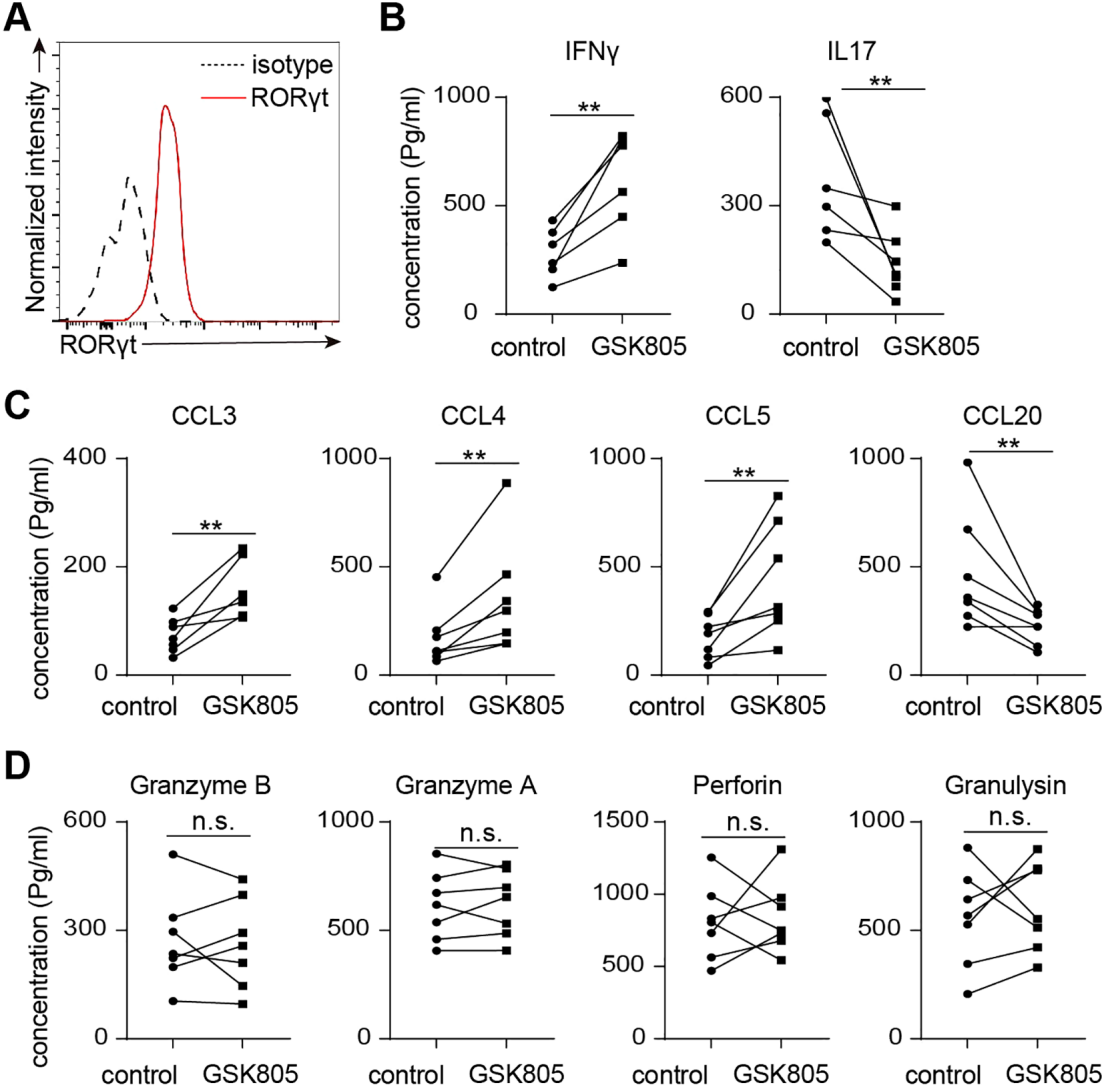
RORγt inhibition leads to reduced IL17 but increased IFNγ production in human MAIT cells. **(A)** Representative flow cytometry profiles showing expression of RORgt by human colon laminal propria MAIT cells. **(B)** 50,000 sorted MAIT cell were cultured with IL-7 and plate-bound anti-CD3 and anti-CD28 antibodies, in the presence of RORgt inhibitor GSK805 or vehicle control, for 48 hours. Shown are the concentrations of IL17 and IFNg measured by multiplex assay. **(C)** The concentrations of chemokines measured by bead-based multiplex assays. **(D)** The concentrations of cytotoxic proteins measured by bead-based multiplex assays. Data are from 7 human individuals. **p<0.01; n.s, not significant.

We used the RORγt inhibitor GSK805 to further investigate the role of RORγt in regulating human MAIT cell function. Flow cytometry analysis revealed that nearly all MAIT cells expressed RORγt (**Figure 6A**). Inhibition of RORγt by GSK805 led to an increase in the production of Th1-related cytokines and chemokines, including IFNγ, CCL3, CCL4, and CCL5, while the production of Th17-related cytokines and chemokines, such as IL17A and CCL20, was significantly reduced (**Figures 6B, C**). Additionally, the production of cytotoxic molecules by human MAIT cells remained unchanged following GSK805 treatment (**Figure 6D**). Together, these data indicate that RORγt inhibits the production of Th1-like cytokines and chemokines while promoting Th17-like cytokines and chemokines, without affecting the production of cytotoxic molecules in human MAIT cells.

### 3.5 Decreased expressions of RORC and IL17 correlate with poorer disease prognosis in colon cancer

**Figure 7:**
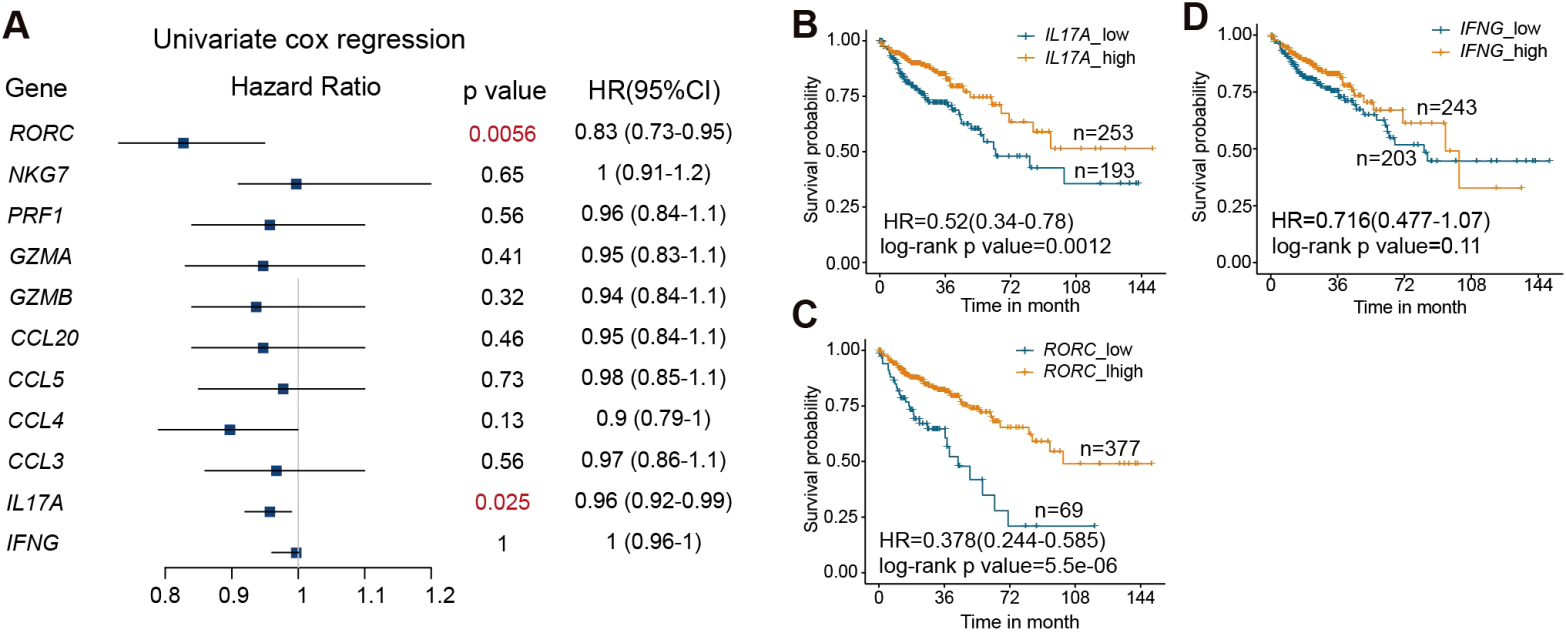
RORC and IL17A expression in the tumor positively correlate with survival in colon cancer patients. **(A)** Correlation between the expression levels of the indicated genes in tumors and mortality of colon cancer patients, by univariate cox regression test. **(B)** Survival curves of colon cancer patients with high and low expression of IL17A. **(C)** Survival curves of colon cancer patients with high and low expression of RORC. **(D)** Survival curves of colon cancer patients with high and low expression of IFNG. Data are from 446 patients from the TCGA-COAD cohorts.

To determine the association of MAIT effector function with disease prognosis, we examine the correlation between the expression of MAIT cell effector genes with patient survival using the gene expression data and clinical information of 446 patients from TCGA-COAD cohorts (32). We first applied a univariate Cox proportional-hazards regression model to assess the correlate of each MAIT cell effector molecule gene with patient mortality. Our results reveal that a lower level of IL17A and RORC in tumors correlates with worse survival in COAD patients (**Figure 7A**). We then separated patients into high-expression and low-expression groups for IL17A, RORC, and IFNG, using a best fit model to determine the cut-off points. Patients with lower expression of IL17A and RORC in tumors exhibited significantly poorer survival compared with those with higher expression of IL17A and RORC (**Figures 7B, C**). In contrast, the levels of IFNG were not associated with prognosis (**Figure 7D**). Together, these data indicate that expression levels of the MAIT17 signature gene IL17A and a positive MAIT17 regulator RORC positively correlate with prognosis of colon cancers.

## 4 Discussion

In this study, we demonstrate that aging drives a shift in both the transcriptomes and functional properties of intestinal MAIT cells, changing from MAIT17 to MAIT1 profiles. Our results show that human MAIT17 and MAIT 1 cells express similar effector gene expression patterns to those found in MAIT cells from young and aged mice. We identified RORγt as an important regulator of MAIT17 versus MAIT1 effector function, and revealed a positive correlation of RORC and IL17 expression with the prognosis of colon cancer (33–36). These results together highlight the dynamic nature of MAIT cell effector function and provide insights into the intricate biology of innate-like T cells.

Aging is a complex process affecting all organs and systems (37). Aging is associated with progressive dysfunction of the adaptive immune system, leading to decreased defense against pathogens and malignancies and impaired vaccine responses (38). The immunodeficiencies occur alongside with inflammaging, characterized by heightened basal level of inflammation in aging bodies (39, 40). In addition, various aging-associated lymphocyte populations with distinctive molecular and functional properties emerge, although their importance remains to be fully understood (41–46). In this study, we revealed found that aging induces drastic changes in the transcriptomes and functional capabilities of mucosal MAIT cells. Notably, MAIT cells from aged mice exhibit comparable proliferative capabilities as those from young, suggesting minimal effects of cellular senescence. Despite an increased proportion of cells with an inactive MAIT cells with aging, MAIT cells exhibiting proliferative and active phenotypes are retained with aging. However, aging is associated with a prominent shift from MAIT17 to MAIT1 function, together with variable alterations in the expressional levels of cytotoxic molecules. While the physiological and pathologic implications of these changes in health and diseases remain to be fully explored by future investigation, our findings highlight the complex effects of aging on innate-lymphocyte dynamics, providing insights into the mechanisms of immune cell aging.

Our work also identified RORγt as an important regulator of MAIT17 versus MAIT1 effector functions in both mice and humans RORγt is a key transcription factor involved in Th17 and group-3 innate lymphoid cell (ILC3) differentiation, and is highly expressed in double positive (DP) thymocytes during early T cell development (47–50). Here using RORγt inhibitors, we explored the role of RORγt in mature MAIT cell function. We found that RORγt is dispensable for the maintenance and proliferation of mature MAIT cells, but it promotes IL17 expression and represses IFNG expression in MAIT cells. MAIT cells in aged mice exhibited lower levels of RORγt compared to those in young mice, which may contribute to a shift from a MAIT17 profile to a MAIT1 profile with aging. Of note, inhibition of RORγt increased IFNγ and Th1-related chemokine expression in MAIT1-like cells from aged mice; however, it was insufficient to induce IFNγ expression in MAIT17-like cells from young mice. Thus, RORγt inhibition can modulate the levels of cytokine and chemokine gene expression, but does not lead to a conversion between MAIT1 and MAIT17 subsets. Our results also indicate that MAIT cells and MAIT17 subset express varying levels of different cytotoxic molecules, which were not affected by RORgt inhibition. Targeting RORgt might offer a viable strategy for selectively altering cytokine and chemokine production of MAIT cells without compromising their cytotoxic activity, potentially influencing the course of certain diseases.

We have also explored the correlation between MAIT cell effector molecules with disease prognosis in colorectal cancers. Analysis of TGCA databases reveal that higher expressions of RORC and IL17 are associated with better prognosis in colorectal cancer patients. IFNG or cytotoxic molecule expression did not show significant correlations with disease prognosis. In healthy humans, IL-17 is produced at basal levels in the intestines, contributing to intestinal homeostasis and epithelial barrier integrity and repair (33, 51, 52). While some studies suggest pro-tumorigenic potential of IL-17, others propose that it may play a protective role (33–36). Our unbiased genomic analysis from TCGA databases revealed a positive correlation between both IL-17 and RORC and improved survival in colorectal cancer patients. However, it remains unclear whether this correlation is due to IL-17 itself or reflects the activity of specific cell populations expressing IL-17 and RORC. Given the abundance of MAIT cells in humans, the decline in MAIT17 profile with aging may contribute to mechanisms of the increased susceptibility to COAD with aging. Future studies to explore the distinctive functions of MAIT1 versus MAIT17 and their effector molecules in disease development and progression would be highly worthwhile. In addition, our focus on colorectal cancer (COAD) was driven by the availability of well-established large gene expression datasets, which provided high-quality data with adequate statistical power. Exploring MAIT cell gene expression profiles in other intestinal disorders and in cancers of different organs represents an important avenue for future research.

Our study has several limitations, including a small sample size of human tissue samples and a relatively narrow focus on intestinal tissue and colorectal adenocarcinoma (COAD). Additionally, our analysis was restricted to MAIT cells and did not encompass other immune cell types. Future research should aim to expand these findings by investigating MAIT cells from various organs and exploring other tissue-resident T cell subsets. Furthermore, because tetramer staining requires live T cells with functional TCRs, we were unable to perform immunofluorescence or immunohistochemical staining to identify mouse MAIT cells in fixed or frozen tissue. Overcoming this technical challenge is essential for revealing the precise locations where MAIT cells accumulate with aging.

We used mouse models to study the effects of aging on MAIT cells, because mouse models allow for controlled conditions with fewer confounding factors. Whether aging has similar effects on MAIT cells at mucosal barriers in humans remain to be elucidated by future investigation. A few recent publications indicate that the frequencies of circulating MAIT cells in the peripheral blood of humans undergo dynamic changes with aging (53, 54). However, exploring physiological aging in humans presents significant challenges, particularly the lack of feasibility identifying a completely healthy elderly population free from chronic inflammatory disorders and other health conditions, especially among the very elderly. Recent work indicates that MAIT cells are highly sensitive to chronic conditions in humans, with their numbers diminished in states such as obesity, diabetes and severe asthma (55, 56). As MAIT cells are still an understudied subset of immune cells, we lack a comprehensive understanding of other conditions that may influence their abundance. Thus, the physiological effects of aging on MAIT cells may be obscured by the prevalence of inflammatory conditions in the elderly. Additionally, obtaining sufficient mucosal tissue from healthy elderly individuals also lacks feasibility, limiting current studies to peripheral blood MAIT cells. Addressing these challenges in human aging research is essential to uncover the precise physiological effects of aging on MAIT cells.

## Data Availability

The datasets presented in this study can be found in online repositories. The names of the repository/repositories and accession number(s) can be found below: E-MTAB-14483 and E-MTAB-14482 (ArrayExpress).
