# Supplementary figures and images for: The effector function of mucosal associated invariant T cells alters with aging and is regulated by RORγt
Source: Front Immunol. 2024 Nov 28;15:1504806. doi: 10.3389/fimmu.2024.1504806 (PMC11634854; doi:10.3389/fimmu.2024.1504806)

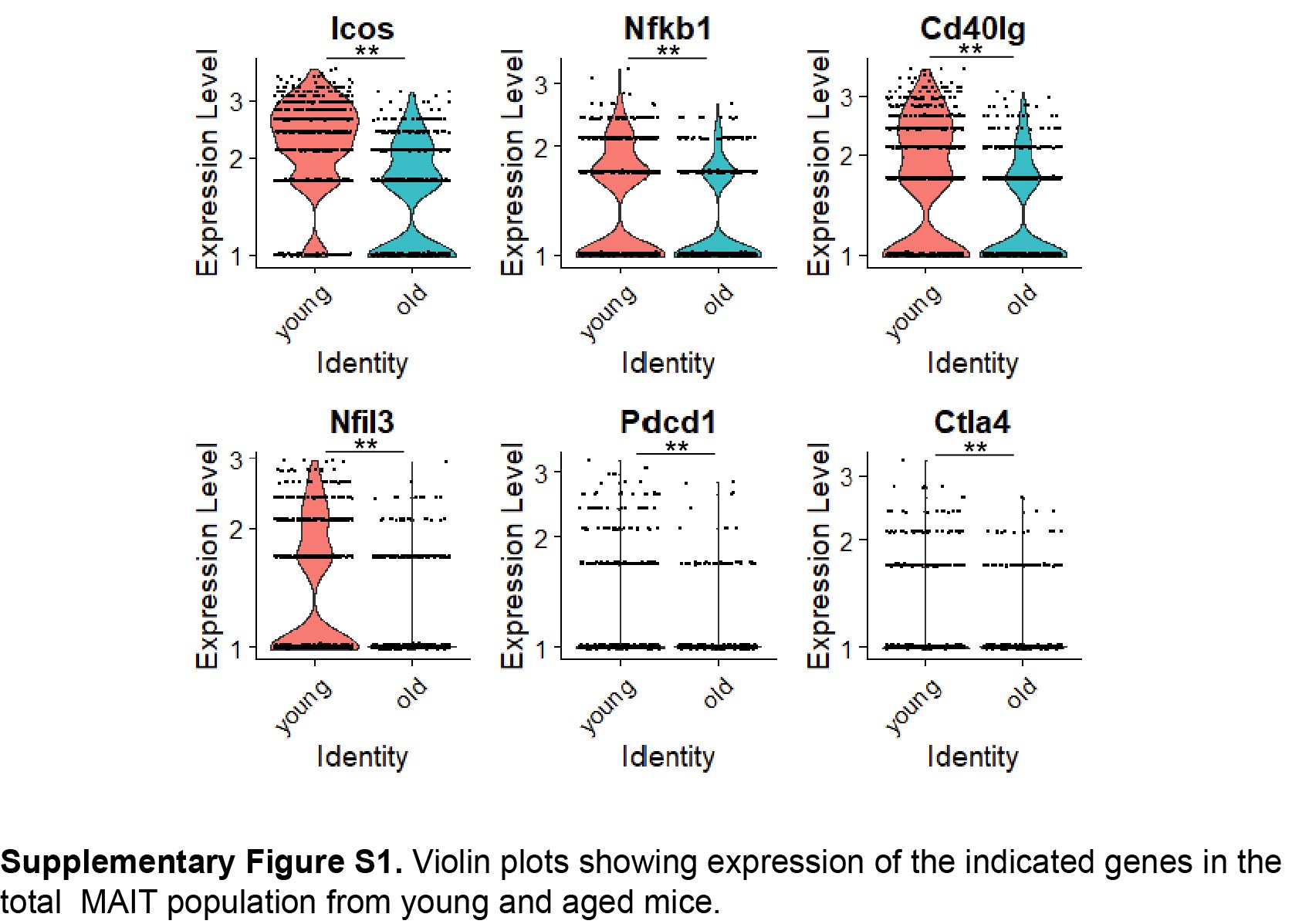

Supplement: Supplementary file 1 [file Image1.jpeg]

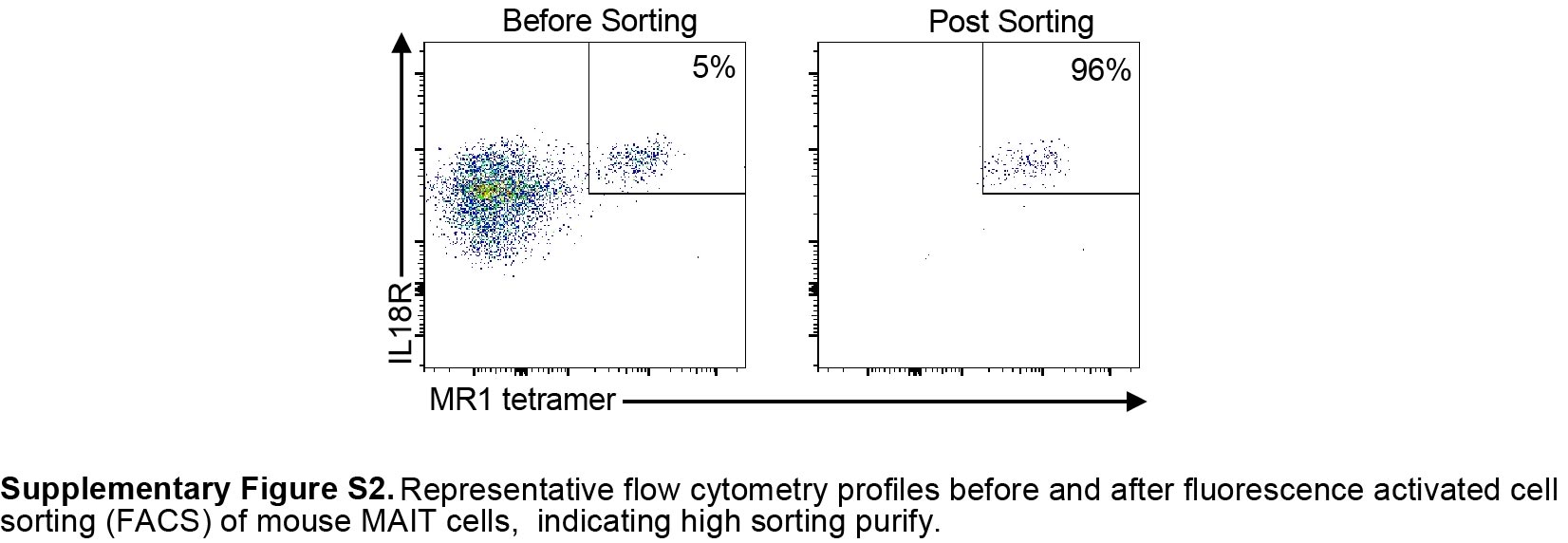

Supplement: Supplementary file 2 [file Image2.jpeg]

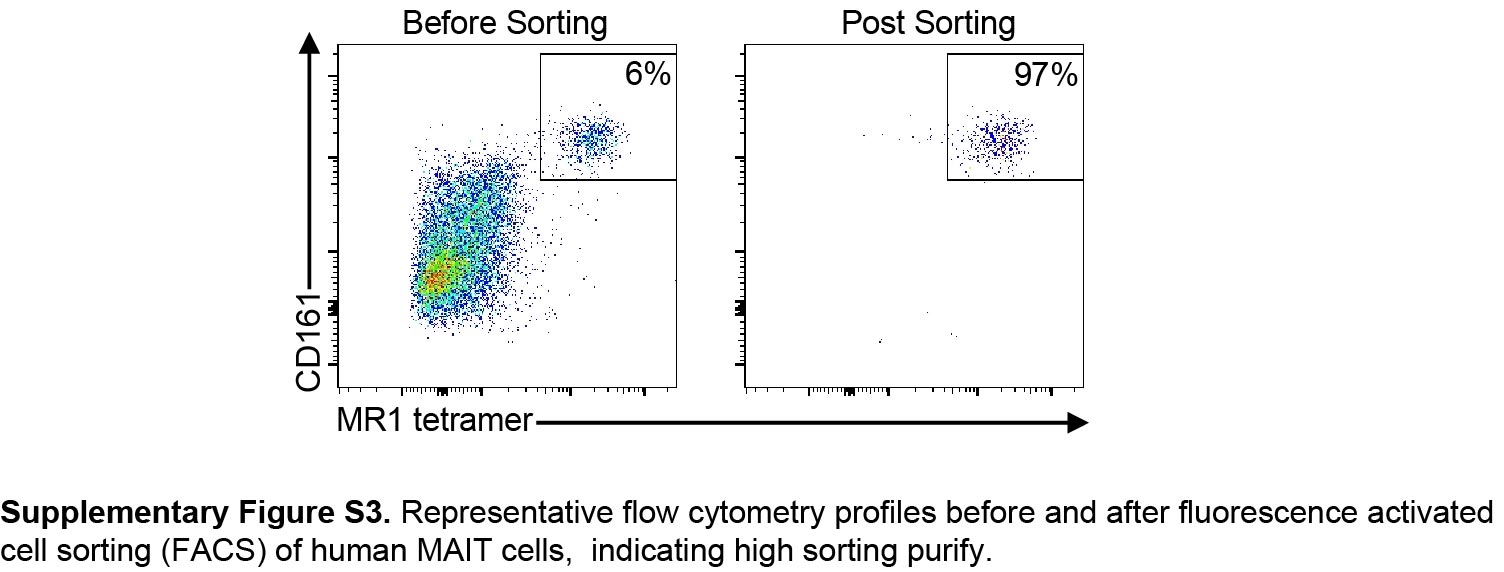

Supplement: Supplementary file 3 [file Image3.jpeg]
